# Investigating the relationship between consultation length and patient experience: a cross-sectional study in primary care

**DOI:** 10.3399/bjgp16X687733

**Published:** 2016-10-25

**Authors:** Natasha Elmore, Jenni Burt, Gary Abel, Frances A Maratos, Jane Montague, John Campbell, Martin Roland

**Affiliations:** Primary Care Unit, Department of Public Health and Primary Care, University of Cambridge School of Clinical Medicine, Cambridge Biomedical Campus, Cambridge.; Primary Care Unit, Department of Public Health and Primary Care, University of Cambridge School of Clinical Medicine, Cambridge Biomedical Campus, Cambridge.; University of Exeter Medical School, St Luke’s Campus, Exeter.; University of Derby, Derby.; University of Derby, Derby.; University of Exeter Medical School, St Luke’s Campus, Exeter.; Primary Care Unit, Department of Public Health and Primary Care, University of Cambridge School of Clinical Medicine, Cambridge Biomedical Campus, Cambridge.

**Keywords:** appointments and schedules, communication, general practice, physician–patient relations, primary health care

## Abstract

**Background:**

Longer consultations in primary care have been linked with better quality of care and improved health-related outcomes. However, there is little evidence of any potential association between consultation length and patient experience.

**Aim:**

To examine the relationship between consultation length and patient-reported communication, trust and confidence in the doctor, and overall satisfaction.

**Design and setting:**

Analysis of 440 videorecorded consultations and associated patient experience questionnaires from 13 primary care practices in England.

**Method:**

Patients attending a face-to-face consultation with participating GPs consented to having their consultations videoed and completed a questionnaire. Consultation length was calculated from the videorecording. Linear regression (adjusting for patient and doctor demographics) was used to investigate associations between patient experience (overall communication, trust and confidence, and overall satisfaction) and consultation length.

**Results:**

There was no evidence that consultation length was associated with any of the three measures of patient experience (*P* >0.3 for all). Adjusted changes on a 0–100 scale per additional minute of consultation were: communication score 0.02 (95% confidence interval [CI] = −0.20 to 0.25), trust and confidence in the doctor 0.07 (95% CI = −0.27 to 0.41), and satisfaction −0.14 (95% CI = −0.46 to 0.18).

**Conclusion:**

The authors found no association between patient experience measures of communication and consultation length, and patients may sometimes report good experiences from very short consultations. However, longer consultations may be required to achieve clinical effectiveness and patient safety: aspects also important for achieving high quality of care. Future research should continue to study the benefits of longer consultations, particularly for patients with complex multiple conditions.

## INTRODUCTION

The impact of consultation length on doctor–patient relationships, workload, and workforce requirements in general practice has long been debated. The 2006–2007 GP workload survey estimated the average consultation length with a GP in the English NHS was 11.7 minutes.^1^^,^^2^ However, this figure was calculated by dividing the total time of recorded appointments by the number of patients seen, and may be an overestimate. A recent study suggested consultation length was nearer to 9 minutes.^3^ The Royal College of General Practitioners has argued that the 10-minute consultation is unsustainable, recommending that primary care appointments should be at least 15 minutes long, inclusive of examinations.^2^ A British Medical Association survey found that 92% of 15 560 GPs felt that 10 minutes for primary care consultations was inadequate.^4^

Research on consultation length and its impact has been conducted in a range of international primary care settings.^5^ In Australia, longer consultations have been associated with older female GPs and, independently, older female patients of higher socioeconomic status.^6^ A Swedish study also found that older patients tended to have longer consultations.^7^ Longer consultations have been associated with increased patient satisfaction.^8^^–^10 However, other research suggests that doctors who have widely varying consultation lengths have a higher proportion rated at least ‘good’ by patients; time may not, in and of itself, represent ‘quality’.^11^ Patient choice of consultation length may lead to both doctors and patients reporting better overall experience and better time management.^12^ There is some evidence that longer consultations are associated with better health outcomes,^13^ including improved hypertension control,^14^ fewer prescriptions,^13^ and better recognition of long-term and psychosocial problems.^13^^,^^15^ A Cochrane Review of changes to consultation length and benefit to patients, doctors, and the healthcare system found that, with more time, doctors did not issue more prescriptions, did not conduct more tests or make more referrals, and patients were not any more satisfied with their care, although doctors had more time for health promotion discussions, and blood pressure may have been measured more frequently. However, the few eligible studies were not of high quality, and the benefits of longer consultations remain unclear.^16^ The authors’ conclusions remained unchanged after an update of the review in 2016.^17^

Little research has considered the relationship between consultation length and patient-reported communication experience. Good communication is an essential component of high-quality health care,^18^ and improving patient experience is at the forefront of recent NHS reforms.^19^

**How this fits in**

Longer consultations have been linked to better health outcomes and are often used as a proxy measure for quality of care in the UK. Patient experience is of increasing interest to policymakers and healthcare professionals, yet little is known about its relationship with consultation length. This study examined the relationship between consultation length and patient experience of communication. Results showed no association between consultation length and patient experience of communication, and some problems may appropriately be addressed in short consultations. However, other elements of quality of care may require longer consultations, especially for patients with multiple or complex problems.

The aim of this study was to investigate the effect of consultation length on patient-reported experience of communication, trust and confidence in the doctor, and overall satisfaction, using videorecordings of doctor–patient consultations to determine consultation length.

## METHOD

Primary care practices were recruited from Devon, Cornwall, Bristol, Dorset and Somerset, Cambridgeshire, Bedford, Luton, and North London. Practices were eligible if they 1) had more than one GP working at least four clinical sessions per week, and 2) had low scores on doctor–patient communication items used in the General Practice Patient Survey (GPPS) (defined as practices below the 25th percentile for mean communication score in the 2009–2010 survey, adjusted for patient case mix).^20^ Low-scoring practices were selected in order to maximise consultations in which patients give low ratings for communication, to meet the aims of the wider research programme.^21^ The authors drew a stratified random sample, stratifying by the communication score banding, GP head count, deprivation index, and geographical location. The authors approached eligible practices in a randomised order until the quota for each stratum was obtained.^22^

All GPs conducting four or more clinical sessions per week were invited to participate. For maximum inclusivity, all patients aged >18 years old attending for a face-to-face consultation with a participating GP were eligible. Recruitment took place face-to-face in the waiting room with study researchers. Patients who lacked capacity to provide written consent, and patients with pre-booked appointments who informed the receptionist that they did not want to be approached, were excluded. Methods by which consultation length data have been collected in previous studies are diverse, and not always robust.^11^^,^^15^^,^^23^^–^25 Videorecording is an accurate and objective measure of consultation length^26^ with no undue impact on participants’ behaviour.^27^^,^^28^ Patients provided prior written consent for their consultation to be videorecorded, confirmed verbally by GPs at the start of the consultation. GPs chose either to leave the camera running continuously, switching it off when a patient declined, or to start each recording as a patient provided verbal consent.

**Figure 1.**
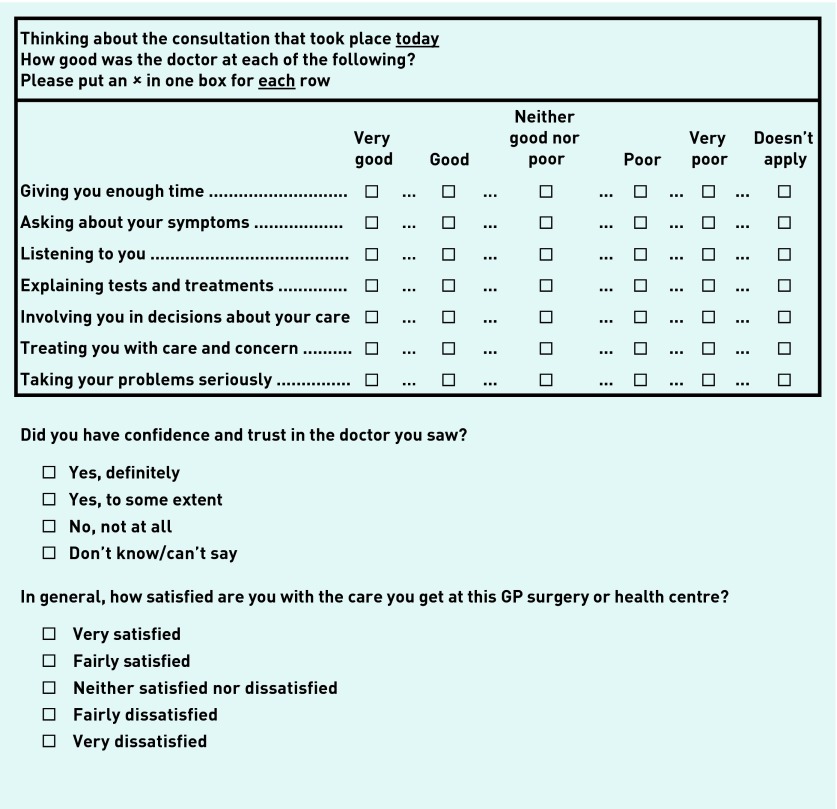
***GP–patient communication, trust, and confidence, and satisfaction items. Items resulted in a linear-scale outcome measure between 0 and 100. ^a^Considered missing data for the purpose of analyses.***

Patients completed a short questionnaire immediately after their consultation, based on the national GPPS^29^ including seven items evaluating communication (Figure 1), the psychometric properties of which have been validated.^30^ As per previous work, the authors calculated a doctor–patient communication score by linearly rescaling responses between 0 and 100 and taking the mean of all responses where four or more informative answers were given.^20^^,^^31^

Basic sociodemographic data were collected (patient age, gender, ethnicity, self-rated health, doctor gender). To ensure accurate estimates of time spent in consultation, the authors calculated consultation length by subtracting the end time of the consultation from its start time, indicated by the doctor asking, ‘What can I do for you today?’ or similar. Examination duration was recorded from when both parties left the field of view and until both returned, provided the audio continued. Examinations taking place on camera (for example, blood pressure testing, temperature observations, weight checks, and ear examinations) were measured from the point at which the examination began until the examination had stopped. Blood tests, urine tests, and the like were considered a ‘bedside’ test and included in the overall consultation duration. Interruptions consisted of telephone calls, entry of an unexpected third party, or the doctor or patient leaving the room. Joint consultations, postnatal checks, incomplete video-questionnaire dyads (that is, videos without linked patient questionnaire), videos missing a clear beginning or end, or otherwise incomplete, were excluded from the final sample.

To detect a moderate relationship with all three patient experience outcomes and time (*R*^2^ = 0.13), with a large effect size and acceptable power (that is, 0.95; with α set at 0.05), the calculated sample size required was 134 videoed consultation dyads. However, as this study involved secondary analyses of data already collected and data from a much larger sample were accessible, disregarding some patient data over others would have been unethical. Therefore the entire sample was used. Linear regression was used to investigate associations between mean communication score, trust and confidence in the GP, and satisfaction, adjusted for patient gender, age, self-rated health, ethnicity, and doctor gender. Bootstrapping was used to obtain confidence intervals (CIs) and *P*-values due to the skewed nature of patient experience scores, clustered by GP to account for the non-independence of observations from individual GPs. Unadjusted and adjusted scores are reported. Sensitivity analyses were conducted to test whether excluding examinations and interruptions had an effect on patient experience. The primary analysis was conducted on total consultation duration (that is, consultation duration as measured, including examination and interruption time).

Shorter consultations may be more acceptable to patients seeing their preferred doctor; some information gathering could be negated, potentially masking the relationship of interest. The authors conducted a sensitivity analysis restricting their sample to patients who expressed a preference for a particular doctor and subsequently adjusted for whether they saw that doctor.

Initial analyses were conducted using sub-items of the communication scale, including item 1: ‘[how good was the doctor at] giving you enough time?’ As these did not show any significant associations, results are not reported.

Non-linear relationships between patient experience and consultation lengths were explored using scatter plots and by adding a quadratic term to the regression models. No evidence of a non-linear relationship was found, therefore results are not shown.

Original data were collected between January 2012 and January 2014. Data were analysed using Stata (version 13.1).

## RESULTS

Of the 741 eligible patients in the original study, 529 patients (71.4%) provided consent and completed a questionnaire. In the final analysis, 440 consultations (45 GPs from 13 primary care practices) were included. Examinations were present in 322 (73%) consultations and 21 (5%) consultations contained interruptions. Owing to the quota sampling strategy, a simple participation rate for practices could not be calculated.

Female patients represented 60% (*n* = 262) of the sample and the most common age category was 65–74 years, comprising one-fifth of the sample (*n* = 86). Patients were predominantly white (91%, *n* = 399) and most reported their health to be ‘good’ or ‘very good’ (Table 1).

**Table 1.** Sociodemographic data

		**Completed questionnaire**	

		***n***	**%**
**Gender**	Male	178	40.5
	Female	262	59.5

**Age, years**	18–24	33	7.5
	25–34	64	14.5
	35–44	52	11.8
	45–54	71	16.1
	55–64	72	16.4
	65–74	86	19.5
	75–84	51	11.6
	≥85	11	2.5

**Self-rated health**	Excellent	37	8.4
	Very good	150	34.1
	Good	151	34.3
	Fair	70	15.9
	Poor	32	7.3

**Ethnicity**	White	399	90.7
	Non-white	41	9.3

**Figure 2.**
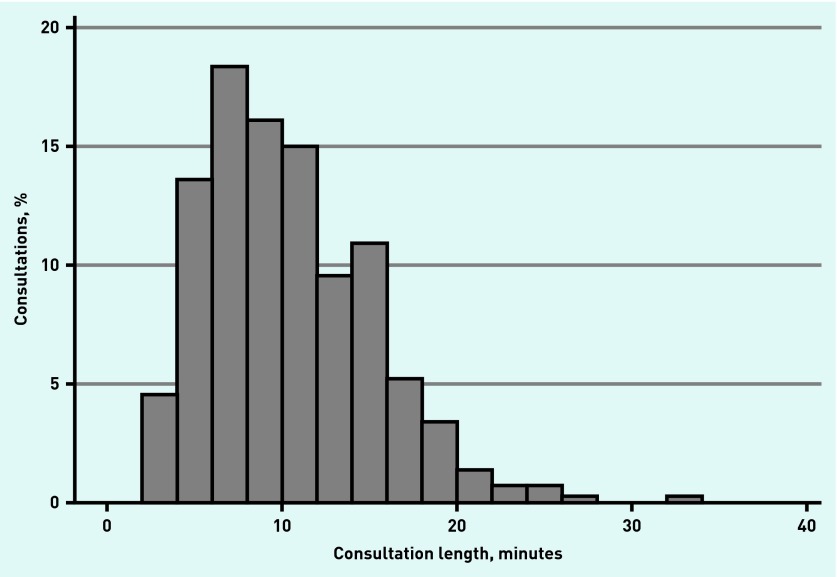
***Distribution of consultation duration, minutes.***

The shortest consultation was 2 minutes 15 seconds and the longest >30 minutes. The distribution of consultation length was skewed (Figure 2) with a greater number of shorter consultations (mean length 10 minutes 22 seconds, standard deviation [SD] 4 minutes 45 seconds). Patient-reported communication scores were highly skewed with 276 (63%) participants reporting a maximum score of 100 (mean score 94.3, SD 10.1). Similarly 396 (90%) and 304 (70%) patients endorsed the highest rating for the two other patient experience items indicating, respectively, that they had definite trust and confidence in their doctor, and that they were very satisfied with their overall care.

There was no evidence of any association between patient-reported communication scores and consultation length either crudely, or when adjusted for patient and doctor characteristics (for each minute of additional consultation length: unadjusted difference 0.06, 95% CI −0.15 to 0.26, *P* = 0.592; adjusted difference 0.02, 95% CI −0.20 to 0.25, *P* = 0.841, Table 2). Although a lack of evidence is not a lack of an effect, the authors noted that CIs were narrow, suggesting that any effect of consultation length, if present, would be small. Patients with poorer self-rated health reported poorer communication scores. There was no evidence that ethnicity or gender of doctor was associated with systematic variation in communication scores, but this may reflect a lack of power as indicated by the relatively wide CIs (Table 2).

**Table 2.** Crude and adjusted mean differences in communication score estimated from linear regression models^a^

		**Unadjusted mean score difference on 0–100 scale (95% CI)**	***P*-value**	**Adjusted mean score difference on 0–100 scale (95% CI)^b^**	***P*-value**
**Length of consultation (per minute)**		0.06 (−0.15 to 0.26)	0.592	0.02 (−0.20 to 0.25)	0.841

**Patient gender**	Male	Reference	0.086	Reference	0.025
	Female	1.94 (−0.27, 4.16)		2.38 (0.30 to 4.47)		

**Patient age**	18–24	Reference		Reference	
	25–34	3.60 (−1.92 to, 9.13)		2.51 (−3.30 to 8.31)	
	35–44	3.26 (−3.03 to 9.56)		3.14 (−3.93 to 10.22)	
	45–54	4.59 (−0.80 to 9.99)		4.50 (−1.51 to 10.51)	
	55–64	5.72 (0.38 to 11.07)	0.003	5.98 (−0.15 to 12.11)	<0.001
	65–74	7.01 (1.74 to 12.28)		7.18 (1.41 to 12.96)	
	75–84	7.03 (1.66 to 12.40)		7.30 (1.25 to 13.35)	
	≥85	8.06 (1.78 to 14.35)		7.44 (0.38 to 14.50)	

**Patient self-rated health**	Excellent	Reference		Reference	
	Very good	−3.31 (−5.18 to −1.44)		−4.02 (−5.94 to −2.10)	
	Fair	−4.60 (−7.10 to −2.10)	0.001	−5.37 (−7.98 to −2.75)	<0.001
	Poor	−3.12 (−5.53 to −0.72)		−5.03 (−8.02 to −2.03)	
	Very poor	−1.83 (−4.83 to 1.17)		−3.49 (−6.79 to −0.19)	

**Patient ethnicity**	White	Reference	0.638	Reference	0.925
	Non-white	0.99 (−3.12 to 5.10)		0.20 (−3.92 to 4.32)	

**Doctor gender**	Male	Reference	0.951	Reference	0.901
	Female	0.08 (−2.52 to 2.68)		−0.16 (−2.75 to 2.42)	

a This is the average difference in score on a 0–100 scale between groups or attributable to an additional minute of consultation length either with or without adjustment for other factors.

b Mean score adjusted for patient gender, age, self-rated health, ethnicity, and doctor gender.

There was no evidence of an association between consultation length and trust and confidence (*P* = 0.681 for adjusted difference, Table 3) or overall satisfaction (*P* = 0.399 for adjusted difference, Table 4). Again, 95% CIs were narrow, indicating that any influence of consultation length on patient-reported trust and confidence was likely to be small.

**Table 3.** Crude and adjusted mean differences in trust and confidence score estimated from linear regression models^a^

		**Unadjusted mean score difference on 0–100 scale (95% CI)**	***P*-value**	**Adjusted mean score difference on 0–100 scale (95% CI)^b^**	***P*-value**
**Length of consultation (per minute)**		0.07 (−0.23 to 0.37)	0.653	0.07 (−0.27 to 0.41)	0.681

**Patient gender**	Male	Reference	0.453	Reference	0.679
	Female	−1.08 (−3.91 to, 1.74)		−0.52 (−2.97 to 1.93)	

**Patient age**	18–24	Reference		Reference	
	25–34	−2.74 (−10.61 to 5.13)		−3.58 (−12.01 to, 4.86)	
	35–44	1.81 (−5.50 to 9.11)		1.30 (−6.58 to 9.18)	
	45–54	1.24 (−6.23 to 8.70)		1.53 (−7.01 to 10.07)	
	55–64	4.05 (−2.51 to 10.62)	0.004	4.25 (−3.32 to 11.82)	0.003
	65–74	5.25 (−0.77 to 11.27)		5.38 (−1.39 to 12.14)	
	75–84	5.61 (−0.50 to 11.73)		6.38 (−0.94 to 13.70)	
	≥85	3.03 (−6.75 to 12.81)		1.74 (−7.29 to 10.77)	

**Patient self-rated health**	Excellent	Reference		Reference	
	Very good	−2.68 (−6.30 to 0.95)		−4.64 (−9.16 to −0.11)	
	Fair	−5.32 (−10.09 to −0.54)	0.163	−7.74 (−13.77 to −1.71)	0.068
	Poor	−3.65 (−8.15 to 0.86)		−7.17 (−12.91 to −1.43)	
	Very poor	−6.46 (−14.44 to 1.52)		−9.33 (−18.10 to −0.57)	

**Patient ethnicity**	White	Reference	0.547	Reference	0.738
	Non-white	2.41 (−5.42 to 10.23)		1.51 (−7.35 to 10.38)	

**Doctor gender**	Male	Reference	0.847	Reference	0.807
	Female	0.42 (−3.84 to 4.67)		0.52 (−3.66 to 4.70)	

a This is the average difference in score on a 0–100 scale between groups or attributable to an additional minute of consultation length either with or without adjustment for other factors.

b Mean score adjusted for patient gender, age, self-rated health, ethnicity, and doctor gender.

**Table 4.** Crude and adjusted mean differences in overall satisfaction score estimated from regression models^a^

		**Unadjusted mean score difference on 0–100 scale (95% CI)**	***P*-value**	**Adjusted mean score difference on 0–100 scale (95% CI)^b^**	***P*-value**
**Length of consultation (per minute)**		−0.12 (−0.45 to 0.20)	0.455	−0.14 (−0.46 to 0.18)	0.399

**Patient gender**	Male	Reference	0.381	Reference	0.689
	Female	−1.35 (−4.37 to 1.67)		−0.54 (−3.21 to 2.12)	

**Patient age**	18–24	Reference		Reference	
	25–34	−0.97 (−9.94 to 7.99)		−1.12 (−10.54 to 8.30)	
	35–44	4.43 (−4.62 to 13.48)		5.40 (−4.25 to 15.04)	
	45–54	−4.78 (−13.84 to 4.28)		−3.80 (−13.04 to 5.45)	
	55–64	4.02 (−5.64 to 13.69)	0.027	5.04 (−4.99 to 15.08)	0.028
	65–74	5.06 (−3.18 to 13.30)		6.28 (−2.11 to 14.67)	
	75–84	4.77 (−4.68 to 14.22)		6.76 (−2.60 to 16.12)	
	≥85	7.58 (−2.48 to 17.63)		8.24 (−1.89 to 18.37)	

**Patient self-rated health**	Excellent	Reference		Reference	
	Very good	−4.32 (−9.64 to 1.00)		−5.13 (−10.89 to 0.62)	
	Fair	−3.99 (−8.76 to 0.78)	0.142	−4.80 (−10.15 to 0.55)	0.232
	Poor	−4.99 (−10.97 to 0.99)		−6.38 (−12.61 to −0.16)	
	Very poor	−5.64 (−12.56 to 1.29)		−5.35 (−13.49 to 2.80)	

**Patient ethnicity**	White	Reference	0.029	Reference	0.023
	Non-white	−4.27 (−8.09 to −0.45)		−5.08 (−9.45 to −0.70)	

**Doctor gender**	Male	Reference	0.700	Reference	0.519
	Female	−0.84 (−5.11 to 3.43)		−1.24 (−5.00 to 2.52)	

a *This is the average difference in score on a 0–100 scale between groups or attributable to an additional minute of consultation length either with or without adjustment for other factors*.

b Mean score adjusted for patient gender, age, self-rated health, ethnicity, and doctor gender.

Sensitivity analyses adjusting for preferred doctor showed that those who saw their preferred doctor reported better communication; however, this had no material impact on the association between consultation length and communication score. Consequently results are not shown. Sensitivity analyses excluding examination and interruption time showed concordant findings (results not presented).

## DISCUSSION

### Summary

This study investigated the relationship between consultation length and patient experience in primary care. The mean consultation length (10 minutes 22 seconds) was less than the national average of 11 minutes 42 seconds^1^^,^^2^ reported in 2007 for GP partners in the English NHS. The consultation length in the current study included only face-to-face contact time, reflecting the time doctors spent directly with the patient; other measures may include time spent reading and recording medical notes. However, consultation length in the current study was slightly longer than that reported elsewhere.^3^^,^^26^^,^^32^ There was no association between consultation length and patient experience of communication, trust and confidence in the doctor, or overall satisfaction. Consistent with previous findings^33^ female patients reported higher communication scores, as did older patients. There was also an effect of patient self-rated health on communication score.

### Strengths and limitations

To the authors’ knowledge, this is the first study that has investigated the association between patient experience of communication and consultation length, measured at encounter level. This association has been investigated previously but analysis took place at session level (that is, surgery sessions booked at 5, 7.5, or 10-minute intervals).^8^ Doctors routinely videorecord consultations for continued professional development and training, but the use of video to collect robust data on consultation length for research is relatively novel, found only in a small number of studies to date.^26^^,^^32^^,^^34^ Consultation length was calculated by the researcher post-consultation, in contrast to the use of appointment systems and other methods.^11^^,^^15^^,^^23^^–^25 A well-validated questionnaire was used immediately after the consultation to collect patient experience and sociodemographic data,^30^ reducing delayed recall effects. CIs were narrow, indicating that the effect of consultation length on outcomes measured is likely to be small.

To align with the broader aims of the research programme,^21^ the authors recruited practices with lower GPPS scores for patient experience of communication, so as to access more low-scoring consultations than typical (in the GPPS, 94% of patients score all communication items as good or very good).^35^ The authors therefore caution that findings from the current study may not be generalisable to all general practices. However, the authors have shown previously that lower-performing practices can include doctors with a wide range of communication scores.^22^ Further, sociodemographic data were broadly representative of patients attending general practices in the wider population and the mean overall communication score was 94.3 points out of 100.

Responses to patient experience questionnaires are often positively skewed^36^^–^39 and there is increasing evidence that patients may be inhibited in criticising their doctors in survey instruments (N Llanwarne *et al*, unpublished data, 2016).^21^^,^^40^^,^^41^ It is therefore possible that the current survey data may not fully reflect consultations that patients find unsatisfactory; this could be more pronounced for shorter consultations.

Reasons for consulting may influence patient experience and the acceptability of consultation length. Such data were not systematically collected in this study, as has been undertaken elsewhere,^42^ and the authors were therefore unable to adjust for these factors.

Reasons for withholding consent were not formally collected but observations made during data collection suggest that these may have included sensitivities around motives for consulting.^43^^–^45 Mental health conditions have been associated with both longer^46^ and shorter consultations,^47^ and therefore the current sample may not be representative of those with more sensitive conditions.

### Comparison with existing literature

Previous research investigating the relationship between consultation length and patient experience outcomes has focused on patient satisfaction, reporting heterogeneous findings. The authors’ findings align with conclusions from a recently updated Cochrane Review that found no relationship between patient satisfaction and consultation length.^16^^,^^17^

Patient-reported experience of communication is associated with patient age, ethnicity, and self-rated health;^33^ the current study suggests that consultation length is not, however, an independent driver of communication or satisfaction, differing from previous research on consultation length and satisfaction.^13^

Patients have also been shown to underestimate the time spent with their doctor^48^ and previous research has suggested that complex consultations require additional time.^49^ The authors’ findings suggest that doctors may already be experts at judging how much time is appropriate on a case-by-case basis, with very short consultations being sufficient in some circumstances and longer consultations provided when required. This is an interpretation consistent with previous literature.^5^

### Implications for research and practice

The authors found no association between patient experience of communication and consultation length. Some consultations may be appropriately short, with both doctor’s and patient’s agenda effectively addressed, for example, where the doctor is dealing with a simple administrative issue or following up a problem with a patient whom they know well. However, longer consultations may be required for maintaining clinical effectiveness and patient safety, which are also important for high-quality care, especially for patients with complex multiple conditions. Future research should consider the benefits of longer consultations across a wider range of practices, and on patient health outcomes, particularly for those with chronic or multimorbid conditions.
